# Learning from big data: are we undertreating older women with high-risk breast cancer?

**DOI:** 10.1038/npjbcancer.2016.19

**Published:** 2016-06-08

**Authors:** Joseph A Sparano, Hyman Muss

**Affiliations:** 1 Montefiore Medical Center, Albert Einstein College of Medicine, Bronx, NY, USA; 2 University of North Carolina, Chapel Hill, NC, USA

There is increasing interest in leveraging ‘big data’ to address important clinical questions, with the ultimate goal of improving human health, including outcomes for those with cancer.^1^ Examples include ‘CancerLinQ’, an initiative developed by the American Society of Clinical Oncology to improve patient care by integrating large volumes of clinical data with analytical computing tools,^2^ and ‘Sentinel’, an initiative developed by the United States (U.S.) Food and Drug Administration to improve drug safety monitoring after regulatory approval of new drugs.^3^ Mining the big data can sometimes lead to big surprises.

In this issue of Breast Cancer, investigators from the U.S. National Cancer Institute leveraged big data that is routinely collected by the federally funded Surveillance Epidemiology and End Results (SEER) program. As described by the authors, SEER is a population-based cancer surveillance program covering ~30% of U.S. residents. Although SEER added breast cancer multi-gene test results to its data collection menu beginning in 2010, including the Genomic Health Oncotype DX Recurrence Score (RS), this analysis required electronically linking the SEER clinical data with the RS data generated between 2004 and 2010 via a collaboration with Genomic Health. The report included 38,568 patients with non-metastatic, estrogen-receptor (ER)-positive, HER2-negative breast cancer, or about 10-fold more than the 3,958 patients originally reported in all prior publications evaluating RS, including five clinical trial cohorts (B14,^4^ *n*=668; B20,^5^ *n*=651; ATAC,^6^ *n*=1017; S8814,^7^ *n*=367; and E2197,^8^ *n*=465), and one prior population-based cohort (*n*=790).^9^ Unadjusted 5-year breast-cancer-specific mortality (BCSM) rates for the low (<18), intermediate (18–30), and high (>30) RS groups were 0.4, 1.4, and 4.4% for node-negative disease (*P*<0.001), and 1.0, 2.3, and 14.3% for node-positive disease (*P*<0.001), respectively. RS remained significantly prognostic in the node-negative cohort when adjusted for age, tumor size, grade, race, and reported adjuvant chemotherapy use. TAILORx (Trial Assigning Individualized Options for Treatment) is a clinical trial designed to determine whether adding chemotherapy to endocrine therapy is beneficial in patients with a mid-range RS of 11–25 and node-negative, ER-positive, HER2-negative disease (NCT00310180).^10^ Using the modified RS categories employed in the TAILORx trial for low (RS<11), intermediate (RS 11–25), and high (RS >25),^10^ 5-year BCSM rates in node-negative disease were 0.4, 0.7, and 3.6%, respectively (*P*<0.001). Although results have not yet been reported for the randomized group with a RS of 11–25, trial participants with a RS<11 treated with endocrine therapy alone had about a 1% risk of distant recurrence at 5 years.^11^ The RxPonder trial (Rx for Positive Node, Endocrine-Responsive Breast Cancer) is also evaluating the benefit of chemotherapy in a higher risk population with 1–3 positive nodes and a RS of 25 or lower (NCT01272037).^12^ Similar to the TAILORx low-risk registry, a recent report from a prospective registry of patients with node-negative, ER-positive, HER2-negative breast cancer reported highly favorable outcomes for patients with a low RS; 5-year distant recurrence rates were 0.5, 2.3, and 4.0% for a RS<18, 18–30, and >30, respectively, associated with corresponding chemotherapy use rates of 1%, 28%, 85%.^13^

Although the results of the SEER analysis do not define the precise RS threshold predictive for adjuvant chemotherapy benefit, they nevertheless provide clear evidence that the assay provides important prognostic information that is generalizable to clinical practice. Several points are worth noting in interpreting these results and placing them in context. First, the end point for the SEER analysis was 5-year BCSM. In prior reports, RS was shown to provide prognostic information for distant metastasis by 10 years, which is generally incurable and leads to death within about 3 years on average.^14^ Thus, the BCSM reflects not only distant metastasis, but also early distant recurrence rapidly leading to death, a clinically meaningful end point. Second, non-breast-cancer mortality was not associated with RS, providing greater confidence that the cause of death was properly classified. Third, although chemotherapy reduces the risk of mostly early recurrences within 5 years of diagnosis,^15^ late recurrence beyond 5 years accounts for at least one-half of recurrences in ER-positive breast cancer,^15–18^ indicating that 5-year BCSM rates will underestimate the true mortality burden in this population.

Beyond the reassuring findings supporting the robustness of the prognostic information provided by RS, another important but surprising finding was the observation of higher 5-year BCSM rates among older women. In the multivariate model including only patients with node-negative disease, an age of 70 years and older was associated with significantly higher 5-year BCSM, which was driven largely by differences in the high RS group. One potential explanation is lower chemotherapy use associated with age. For the node-negative population in this cohort who had a high RS, chemotherapy use was reported in 78%, 75%, 74%, 57%, 56%, and 32% of patients less than 40, 40–49, 50–59, 60–69, 70–79, and 80 years or older, respectively. For patients with a node-positive disease and a high RS, chemotherapy use was reported in 52% of patients 70–79, and 50% of patients >80 years of age. These findings are particularly concerning in view of the previous data, likewise showing poorer breast-cancer-specific survival for older women with early-stage breast cancer.^19^ Although chemotherapy use was likely underreported in the SEER analysis, the findings again support the known age bias against chemotherapy use in older women, even in those at high risk of recurrence who could potentially derive greater benefit from therapy.^20^

Breast cancer is a disease of aging, with a threefold higher risk for women older than 70 years compared with age 50–59 years.^21^ Owing to increasing life expectancy^22^ and increasing incidence with advancing age, a 30% increase in the overall incidence of breast cancer is expected by the year 2030, driven largely by a 57% increase in women 65 years or older.^23^ Age alone should not be used to make adjuvant treatment decisions, as many older women in their 70 and 80s have life expectancies far in excess of 5 years, the follow-up time reported in this SEER-based study. For example, the life expectancy of a women in average health at 70, 75, and 80 years is 12, 9, and 7 years, respectively.^24^ In addition, older women with early breast cancer in good health appear to derive the same benefits from chemotherapy as in younger women,^25^ although there is greater toxicity.^26^ Broader use of validated online models that accurately estimate life expectancy, such as ePrognosis (http://eprognosis.ucsf.edu/index.php), may assist clinicians when formulating adjuvant treatment plans for older patients.

In conclusion, the findings from this population-based study provide additional evidence supporting the clinical validity of the prognostic information provided by the 21-gene RS assay, indicating that it is robust and provides clinically meaningful information that is generalizable to real-world clinical practice. The clinical utility of the assay will likely be further refined once the results of the TAILORx and RxPonder trials are available. In the interim, given the paucity of elderly women enrolled in prospective clinical trials,^27^ the findings from this study provide important information suggesting that greater chemotherapy use in elderly patients with high-risk breast cancer could substantially reduce early-breast-cancer mortality, if used in appropriately selected patients and in accordance with currently recommended guidelines.^27^

## References

[bib1] Kantarjian, H. & Yu, P. P. Artificial intelligence, big data, and cancer. JAMA Oncol. 1, 573–574 (2015). 26181906 10.1001/jamaoncol.2015.1203

[bib2] Shah, A., Stewart, A. K., Kolacevski, A., Michels, D. & Miller, R. Building a rapid learning health care system for oncology: why CancerLinQ collects identifiable health information to achieve its vision. J. Clin. Oncol. 34, 756–763 (2016). 26755519 10.1200/JCO.2015.65.0598

[bib3] Kuehn, B. M. FDA's foray into big data still maturing. JAMA 315, 1934–1936 (2016). 27097048 10.1001/jama.2016.2752

[bib4] Paik, S. et al. A multigene assay to predict recurrence of tamoxifen-treated, node-negative breast cancer. N. Engl. J. Med. 351, 2817–2826 (2004). 15591335 10.1056/NEJMoa041588

[bib5] Paik, S. et al. Gene expression and benefit of chemotherapy in women with node-negative, estrogen receptor-positive breast cancer. J. Clin. Oncol. 24, 3726–3734 (2006). 16720680 10.1200/JCO.2005.04.7985

[bib6] Sgroi, D. C. et al. Prediction of late distant recurrence in patients with oestrogen-receptor-positive breast cancer: a prospective comparison of the breast-cancer index (BCI) assay, 21-gene recurrence score, and IHC4 in the TransATAC study population. Lancet Oncol. 14, 1067–1076 (2013). 24035531 10.1016/S1470-2045(13)70387-5 PMC3918681

[bib7] Albain, K. S. et al. Adjuvant chemotherapy and timing of tamoxifen in postmenopausal patients with endocrine-responsive, node-positive breast cancer: a phase 3, open-label, randomised controlled trial. Lancet 374, 2055–2063 (2009). 20004966 10.1016/S0140-6736(09)61523-3 PMC3140679

[bib8] Goldstein, L. J. et al. Prognostic utility of the 21-gene assay in hormone receptor-positive operable breast cancer compared with classical clinicopathologic features. J. Clin. Oncol. 26, 4063–4071 (2008). 18678838 10.1200/JCO.2007.14.4501 PMC2654377

[bib9] Habel, L. A. et al. A population-based study of tumor gene expression and risk of breast cancer death among lymph node-negative patients. Breast Cancer Res. 8, R25 (2006). 16737553 10.1186/bcr1412 PMC1557737

[bib10] Sparano, J. A. & Paik, S. Development of the 21-gene assay and its application in clinical practice and clinical trials. J. Clin. Oncol. 26, 721–728 (2008). 18258979 10.1200/JCO.2007.15.1068

[bib11] Sparano, J. A. et al. Prospective validation of a 21-gene expression assay in breast cancer. N. Engl. J. Med. 373, 2005–2014 (2015). 26412349 10.1056/NEJMoa1510764 PMC4701034

[bib12] Wong, W. B., Ramsey, S. D., Barlow, W. E., Garrison, L. P. Jr. & Veenstra, D. L. The value of comparative effectiveness research: projected return on investment of the RxPONDER trial (SWOG S1007). Contemp. Clin. Trials 33, 1117–1123 (2012). 22981891 10.1016/j.cct.2012.08.006 PMC3486702

[bib13] Stemmer, S. M. et al. Real-life analysis evaluating 1594 N0/Nmic breast cancer patients for whom treatment decisions incorporated the 21-gene recurrence score result: 5-year KM estimate for breast cancer specific survival with recurrence score results ≤30 is >98%. Cancer Res. 76, P5-08-02 (2015).

[bib14] Tevaarwerk, A. J. et al. Survival in patients with metastatic recurrent breast cancer after adjuvant chemotherapy: little evidence of improvement over the past 30 years. Cancer 119, 1140–1148 (2013). 23065954 10.1002/cncr.27819 PMC3593800

[bib15] Early Breast Cancer Trialists' Collaborative G, et al. Comparisons between different polychemotherapy regimens for early breast cancer: meta-analyses of long-term outcome among 100,000 women in 123 randomised trials. Lancet 379, 432–444 (2012). 22152853 10.1016/S0140-6736(11)61625-5 PMC3273723

[bib16] Saphner, T., Tormey, D. C. & Gray, R. Annual hazard rates of recurrence for breast cancer after primary therapy. J. Clin. Oncol. 14, 2738–2746 (1996). 8874335 10.1200/JCO.1996.14.10.2738

[bib17] Sparano, J. A. et al. Long-term follow-up of the E1199 phase III trial evaluating the role of taxane and schedule in operable breast cancer. J. Clin. Oncol. 33, 2353–2360 (2015). 26077235 10.1200/JCO.2015.60.9271 PMC4500829

[bib18] Colleoni, M. et al. Annual hazard rates of recurrence for breast cancer during 24 years of follow-up: results from the international breast cancer study group trials I to V. J. Clin. Oncol. 34, 927–935 (2016). 26786933 10.1200/JCO.2015.62.3504 PMC4933127

[bib19] Smith, B. D. J. et al. Improvement in breast cancer outcomes over time: Are older women missing out? J. Clin. Oncol. 29, 4647–4653 (2011). 22067407 10.1200/JCO.2011.35.8408

[bib20] Muss, H. B. Adjuvant treatment of elderly breast cancer patients. Breast 16 Suppl 2, S159–S165 (2007). 17728133 10.1016/j.breast.2007.07.026

[bib21] Siegel, R. L., Miller, K. D. & Jemal, A. Cancer statistics, 2016. CA Cancer J. Clin. 66, 7–30 (2016). 26742998 10.3322/caac.21332

[bib22] Shachar, S. S., Hurria, A. & Muss, H. B. Breast cancer in women older than 80 years. J. Oncol. Pract. 12, 123–132 (2016). 26869650 10.1200/JOP.2015.010207

[bib23] Smith, B. D., Smith, G. L., Hurria, A., Hortobagyi, G. N. & Buchholz, T. A. Future of cancer incidence in the United States: burdens upon an aging, changing nation. J. Clin. Oncol. 27, 2758–2765 (2009). 19403886 10.1200/JCO.2008.20.8983

[bib24] Cho, H. et al. Comorbidity-adjusted life expectancy: a new tool to inform recommendations for optimal screening strategies. Ann. Intern. Med. 159, 667–676 (2013). 24247672 10.7326/0003-4819-159-10-201311190-00005

[bib25] Muss, H. B. et al. Adjuvant chemotherapy in older and younger women with lymph node-positive breast cancer. JAMA 293, 1073–1081 (2005). 15741529 10.1001/jama.293.9.1073

[bib26] Muss, H. et al. Toxicity of older and younger patients treated with intensive adjuvant chemotherapy for node-positive breast cancer: The CALGB experience. J. Clin. Oncol. 24, 559 (2006). 10.1200/JCO.2007.10.9710 17704418

[bib27] Wildiers, H. et al. Management of breast cancer in elderly individuals: recommendations of the International Society of Geriatric Oncology. Lancet Oncol. 8, 1101–1115 (2007). 18054880 10.1016/S1470-2045(07)70378-9

